# Supplementary material for: Metabolic Adjustment of High Intertidal Alga Pelvetia canaliculata to the Tidal Cycle Includes Oscillations of Soluble Carbohydrates, Phlorotannins, and Citric Acid Content
Source: Int J Mol Sci. 2023 Jun 25;24(13):10626. doi: 10.3390/ijms241310626 (PMC10341635; doi:10.3390/ijms241310626)
Supplement: Supplementary file 1 [file ijms-24-10626-s001.zip › ijms-2454242-supplementary.pdf]

Metabolic adjustment of high intertidal alga *Pelvetia canaliculata* to the tidal cycle includes oscillations of soluble carbohydrates, phlorotannins, and citric acid content.

Renata Islamova [1], Nikolay Yanshin [1], Elizaveta Zamyatkina [1], Ekaterina Gulk [1], Ekaterina Zuy [1], Susan Billig [2], Claudia Birkemeyer [2,3] and Elena Tarakhovskaya [1,4,*]

[1] Department of Plant Physiology and Biochemistry, Faculty of Biology, Saint Petersburg State University, Universitetskaya nab., 7/9, Saint Petersburg 199034, Russia.

[2] Faculty of Chemistry and Mineralogy, Leipzig University, Linnestr. 3, 04103 Leipzig, Germany.

[3] German Center for Integrative Biodiversity Research (iDiv) Halle-Leipzig-Jena, 04103 Leipzig, Germany.

[4] Vavilov Institute of General Genetics, Saint Petersburg Branch, Russian Academy of Science, Universitetskaya nab., 7/9, Saint Petersburg 199034, Russia.

[*] Correspondence: e.tarahovskaya@spbu.ru, elena.tarakhovskaya@gmail.com

Table S1. Retention times (RT), calculated retention indexes (RI) and content of compounds detected in *Pelvetia canaliculata*. Presented are identified metabolites and chemically classified unknowns. All values of metabolite content represent the means of 5 replicates ± standard deviation (absence of the value means that only relative quantification was made this compound). **HW** – high water, **ET** – ebb tide, **LW** – low water; **RT** – rising tide. Abundant metabolites are marked with bold font.

No.	Analyte	RT (min)	RI	Quantification, *m/z* ± 0.5	Metabolite content, µmol/g dry weight			
					HW	ET	LW	RT
1	Glyoxylic acid	9.37	1090	218	-	-	-	-
2	Oxalic acid	9.51	1097	190	-	-	-	-
3	Valine (2TMS)	11.28	1185	218	0.09±0.02	0.09±0.024	0.04±0.009	0.04±0.008
4	Urea	11.94	1217	189	-	-	-	-
5	Ethanolamine	12.36	1238	174	-	-	-	-
6	Phosphoric acid	12.52	1246	299	-	-	-	-
7	Leucine (2TMS)	12.52	1247	158	0.06±0.01	0.08±0.01	0.03±0.008	0.02±0.004
8	**Glycerol**	12.60	1250	218	2.54±0.99	2.57±0.67	0.55±0.11	1.16±0.26
9	Isoleucine (2TMS)	13.00	1270	218	0.06±0.01	0.07±0.01	0.03±0.01	0.03±0.004

10	Proline	13.04	1272	142	0.11±0.02	0.07±0.02	0.02±0.008	0.02±0.004
11	Glycine (3TMS)	13.22	1284	248	0.03±0.008	0.01±0.005	0.08±0.002	0.006±0.002
12	Succinic acid	13.52	1296	247	0.03±0.004	0.03±0.007	0.06±0.007	0.02±0.004
13	Glyceric acid	13.82	1312	292	-	-	-	-
14	Fumaric acid	14.37	1341	245	0.0013±0.0004	0.0019±0.0002	0.0031±0.0004	0.0015±0.0003
15	Serine (3TMS)	14.47	1346	218	0.12±0.03	0.07±0.02	0.06±0.01	0.04±0.01
16	Threonic acid-1,4-lactone	14.74	1360	247	-	-	-	-
17	Threonine (3TMS)	15.02	1375	291	0.08±0.01	0.10±0.01	0.05±0.007	0.03±0.007
18	2-deoxytetronic acid	16.04	1428	233	-	-	-	-
19	Citramalic acid	16.74	1464	247	-	-	-	-
20	Malic acid	17.18	1487	233	0.0076±0.0005	0.0068±0.0007	0.0064±0.0012	0.0032±0.0004
21	Threitol	17.42	1499	217	-	-	-	-
22	Pentadecane	17.45	1500	71	-	-	-	-
23	Erythritol	17.57	1507	307	-	-	-	-
24	Salicylic acid	17.58	1508	267	0.016±0.002	0.012±0.004	0.121±0.036	0.009±0.002
25	Pyroglutamic acid	17.67	1517	156	-	-	-	-
26	Aspartic acid (3TMS)	17.80	1519	232	0.055±0.013	0.031±0.007	0.025±0.007	0.012±0.003
27	Erythronic acid	18.25	1543	292	-	-	-	-
28	**Threonic acid**	18.61	1562	292	-	-	-	-
29	Pyrogallol	19.35	1600	342	-	-	-	-
30	3-Hydroxy-3-methylglutaric acid	19.38	1602	247	-	-	-	-
31	5-hydroxypipecolic acid	19.45	1606	244	-	-	-	-
32	Glutamic acid (3TMS)	19.75	1621	246	0.28±0.05	0.35±0.06	0.22±0.03	0.09±0.02
33	Phenylalanine (2TMS)	19.77	1622	192	0.05±0.01	0.18±0.03	0.05±0.01	0.03±0.01

34	Phloroglucinol	20.22	1646	342	0.25±0.04	0.31±0.04	0.49±0.05	0.47±0.06
35	Unknown pentose	20.42	1656	103	-	-	-	-
36	Arabinose	20.56	1663	103	0.037±0.001	0.054±0.002	0.064±0.001	0.079±0.001
37	Hydroxypyrimidine [illegible]	20.67	1669	358	-	-	-	-
38	Ribose	20.83	1678	103	-	-	-	-
39	α-Aminoadipic acid	20.96	1684	260	-	-	-	-
40	Unknown pentitol	21.14	1694	103	-	-	-	-
41	Unknown pentitol	21.32	1704	217	-	-	-	-
42	Isocitric lactone	21.36	1706	157	-	-	-	-
43	Arabitol	21.58	1718	307	-	-	-	-
44	Fucose	21.63	1721	160	-	-	-	-
45	Aconitic acid	22.14	1750	229	0.29±0.04	0.22±0.04	0.25±0.04	0.05±0.01
46	Tetrahydroxybenzene	22.25	1756	430	-	-	-	-
47	Lyxonic acid	22.34	1761	292	-	-	-	-
48	Glutamine (3TMS)	22.54	1772	156	0.24±0.02	0.29±0.02	0.04±0.02	0.03±0.01
49	Unknown sugar	22.61	1776	319	-	-	-	-
50	Unknown sugar	23.06	1802	217	-	-	-	-
51	Ornithine	23.26	1813	142	-	-	-	-
52	**Citric acid**	23.37	1820	465	32.13±1.56	30.87±4.79	18.50±2.90	11.37±2.82
53	Isocitric acid	23.39	1821	245	-	-	-	-
54	Unknown sugar	23.50	1827	204	-	-	-	-
55	Homogentisic acid	23.58	1832	384	-	-	-	-
56	Phytol	23.66	1837	123	-	-	-	-
57	Dehydroascorbic acid [illegible]	23.74	1841	173	-	-	-	-

58	Myristic acid	23.84	1847	285	-	-	-	-
59	Unknown fatty alcohol	24.09	1862	123	-	-	-	-
60	**Fructose**	24.21	1869	364	0.16±0.01	0.32±0.12	1.19±0.30	0.69±0.17
61	Mannose	24.45	1883	319	0.006±0.001	0.015±0.004	0.028±0.004	0.030±0.007
62	Galactose	24.51	1887	319	0.05±0.008	0.07±0.03	0.17±0.03	0.10±0.02
63	Glucose	24.62	1893	319	0.09±0.01	0.35±0.07	0.21±0.07	0.53±0.14
64	Lysine (4TMS)	25.19	1928	156	0.02±0.001	0.02±0.002	0.02±0.002	0.02±0.001
65	Tyrosine (3TMS)	25.32	1936	354	-	-	-	-
66	**Mannitol**	25.35	1938	421	117± 21	169±10	186±11	119±30
67	Dulcitol	25.43	1943	319	-	-	-	-
68	Ascorbic acid	25.54	1950	332	-	-	-	-
69	Pentadecanoic acid	25.55	1951	299	-	-	-	-
70	Galacturonic acid	25.75	1963	333	-	-	-	-
71	Unknown sugar	25.79	1966	361	-	-	-	-
72	Ononitol	25.96	1976	318	-	-	-	-
73	Gluconic acid	26.24	1994	333	-	-	-	-
74	Trihydroxybenzoic acid	26.31	1998	443	-	-	-	-
75	Scillo-Inositol	26.70	2024	318	-	-	-	-
76	Unknown sugar	26.88	2036	217	-	-	-	-
77	Unknown sugar	26.96	2041	305	-	-	-	-
78	Palmitic acid	27.02	2045	313	-	-	-	-
79	Unknown polyol	27.16	2055	333	-	-	-	-
80	Myo-Inositol	27.62	2085	318	0.03±0.001	0.03±0.001	0.04±0.002	0.04±0.003
81	Unknown polyol	27.62	2085	259	-	-	-	-

82	Unknown polyol	28.07	2114	307	-	-	-	-
83	**Unknown polyol**	28.31	2130	259	-	-	-	-
84	**Unknown polyol**	29.19	2188	231	-	-	-	-
85	**Volemitol**	29.61	2218	421	-	-	-	-
86	Oleic acid	29.80	2231	339	-	-	-	-
87	Unknown polyol	29.88	2237	217	-	-	-	-
88	Unknown sugar	29.97	2243	204	-	-	-	-
89	Stearic acid	30.00	2246	341	-	-	-	-
90	Unknown disaccharide	30.19	2260	361	-	-	-	-
91	Difucol	30.55	2282	682	-	-	-	-
92	Arachidonic acid	31.63	2363	79	-	-	-	-
93	Diphlorethol	32.03	2392	610	-	-	-	-
94	Glycerol-1-myristate	32.05	2394	343	-	-	-	-
95	Unknown sugar	32.66	2440	217	-	-	-	-
96	Sucrose	35.08	2630	437	0.03±0.008	0.18±0.05	0.13±0.04	0.46±0.10
97	Lactose	35.68	2679	319	0.015±0.005	0.062±0.021	0.021±0.009	0.019±0.003
98	**Trehalose**	36.33	2732	361	-	-	-	-
99	Unknown sugar	36.59	2754	361	-	-	-	-
100	Unknown sugar	36.69	2762	204	-	-	-	-
101	Unknown polyol	36.82	2775	204	-	-	-	-
102	Lactitol	36.91	2780	361	-	-	-	-
103	Tetracosenoic acid	37.10	2796	423	-	-	-	-
104	Squalene	37.21	2805	81	-	-	-	-
105	Maltitol	37.36	2816	204	-	-	-	-

106	**Unknown polyol**	37.86	2858	361	-	-	-	-
107	**Tocopherol δ**	38.14	2880	474	-	-	-	-
108	Unknown sugar	38.25	2890	319	-	-	-	-
109	Unknown sugar	38.40	2901	319	-	-	-	-
110	Unknown sugar	38.55	2914	217	-	-	-	-
111	Unknown sugar	38.85	2938	204	-	-	-	-
112	Tocopherol β	39.07	2957	488	-	-	-	-
113	Unknown sugar	39.16	2964	204	-	-	-	-
114	Tocopherol γ	39.19	2967	488	-	-	-	-
115	Unknown polyol	39.40	2984	204	-	-	-	-
116	Unknown sugar	39.51	2992	361	-	-	-	-
117	Unknown sugar	39.56	2996	204	-	-	-	-
118	Unknown sugar	39.64	3003	361	-	-	-	-
119	**Unknown sugar**	40.56	3078	361	-	-	-	-
120	Tocopherol α	40.69	3089	502	-	-	-	-
121	Cholesterol	40.76	3095	329	-	-	-	-
122	Unknown sterol	41.19	3130	255	-	-	-	-
123	Unknown sterol	41.66	3168	129	-	-	-	-
124	Unknown sterol	41.77	3177	129	-	-	-	-
125	Unknown polyol	41.92	3190	205	-	-	-	-
126	**Fucosterol**	42.71	3254	484	-	-	-	-
127	Unknown sterol	42.85	3266	129	-	-	-	-
128	Unknown trisaccharide	46.06	3529	361	-	-	-	-
129	Unknown trisaccharide	46.27	3546	361	-	-	-	-

130	Unknown trisaccharide	48.25	3708	361	-	-	-	-
131	Unknown trisaccharide	48.35	3716	361	-	-	-	-
132	Unknown trisaccharide	48.42	3722	361	-	-	-	-
